# Myocardial intramural course may reduce left ventricular ejection fraction of patients suffering from coronary heart disease

**DOI:** 10.3389/fcvm.2025.1451173

**Published:** 2025-02-27

**Authors:** Shi Miaomiao, Zheng Jiaqi, Li Xiaomeng, Li Shanshan, Wang Jie, Liu Kaicheng, Jia Mei, Su Ming

**Affiliations:** ^1^ Department of Clinical Laboratory, Peking University People’s Hospital, Beijing, China; ^2^ Institute of Medical Technology, Peking University Health Science Center, Peking University, Beijing, China; ^3^ State Key Laboratory of Vascular Homeostasis and Remodeling, Peking University, Beijing, China

**Keywords:** myocardial intramural course, coronary heart disease, cardiac function, clinical analysis, imaging test

## Abstract

**Background:**

Myocardial intramural course (MIC), a benign anatomical lesion, is an abnormal anatomical structure formed due to abnormal blood vessel routing. An increasing number of studies indicate that MIC is associated with coronary heart disease (CHD). However, it remains unclear whether MIC contributes to cardiac function impairment in patients with CHD. Thus, this study is to observe the association between MIC and cardiac function in patients with CHD.

**Methods:**

All participants were recruited from the Department of Cardiology, Peking University People's Hospital from August 2022 to September 2023. A total of 126 patients were diagnosed with MIC by coronary angiography and/or coronary CT angiography. Among them, a total of 39 patients diagnosed with MIC and CHD were enrolled in the MIC-CHD group. Sixty cases of monthly stratified CHD patients were randomly selected, into the CHD group as controls.

**Results:**

The left ventricular ejection fraction (LVEF) of patients in the MIC-CHD group was lower than that in the CHD group (0.62 vs. 0.67, *p* = 0.0153). LVEF in patients of MIC-CHD was negatively correlated with the systolic stenosis degree of mural coronary artery (MCA) (*r* = −0.6474, *p* = 0.0123) and MIC length (*r* = −0.5712, *p* = 0.0414).

**Conclusions:**

The combination of MIC in patients with CHD may contribute to the reduction of LVEF, whereas MIC length and the systolic stenosis degree of MCA were negatively correlated with LVEF.

## Introduction

The coronary artery running through epicardial adipose tissue is often covered in part with myocardial tissue. This structure is regarded as a myocardial intramural course (MIC) (1, 2). Myocardial muscle bridges are an incidental finding at angiography or postmortem examination in a large number of individuals (3). During the systolic phase, the myocardium surrounding the blood vessels will compress the ectopic vessel, known as the milking effect, resulting in negative effects. Systolic compression of coronary arteries results in limiting coronary blood flow and filling, which leads to stenocardia, myocardial ischemia, and even sudden cardiac death in some severe cases (4). The compression of the neighbor myocardium on the segment of the coronary artery inevitably leads arterial wall shear stress to change, which then acts as a proatherogenic event affecting the endothelium of the arterial segment proximal to the MIC (5, 6), promoting the release of endothelial active factors, cell proliferation, platelet aggregation and adhesion, and formation of proximal coronary atherosclerosis (7, 8).

Coronary heart disease (CHD) has been the leading cause of morbidity and mortality worldwide and is characterized as a chronic immunoinflammatory, fibroproliferative disease that is fueled by lipids (9). Major risk factors resulting in CHD include smoking, hyperlipidemia, hypertension, diabetes mellitus, age, obesity, a sedentary lifestyle, a family history of early onset of CHD, and new negative factors such as diseases that increase systemic inflammation, the inflammasome, and maternal and childhood factors (10). The mechanism of insufficient blood supply and cardiac hypoxia is the narrowing or blocking of the coronary arteries by atherosclerosis (11, 12), resulting in chest distress, angina, and even acute myocardial infarction in some severe cases (13). With the deepening of research, more causes of CHD have been discovered, MIC included (14, 15). Thus, MIC may be one of the causes of cardiac function change in patients with CHD.

Because of the low detection rate of MIC and the inconspicuous myocardial ischemia symptoms in most patients with MIC, and common risk factors for CHD including age, dyslipidemia, hypertension, smoking, diabetes, obesity, and family history are numerous, few clinicians pay sufficient attention to MIC and the studies on MIC-CHD (16–18). However, a few studies have reported that some patients with sudden cardiac death did not die from myocardial infarction caused by coronary atherosclerosis, but rather related to MIC. Therefore, the effect of myocardial intramural courses on cardiac function in patients with CHD should be paid more attention.

## Methods

### Subjects

**Figure 1:**
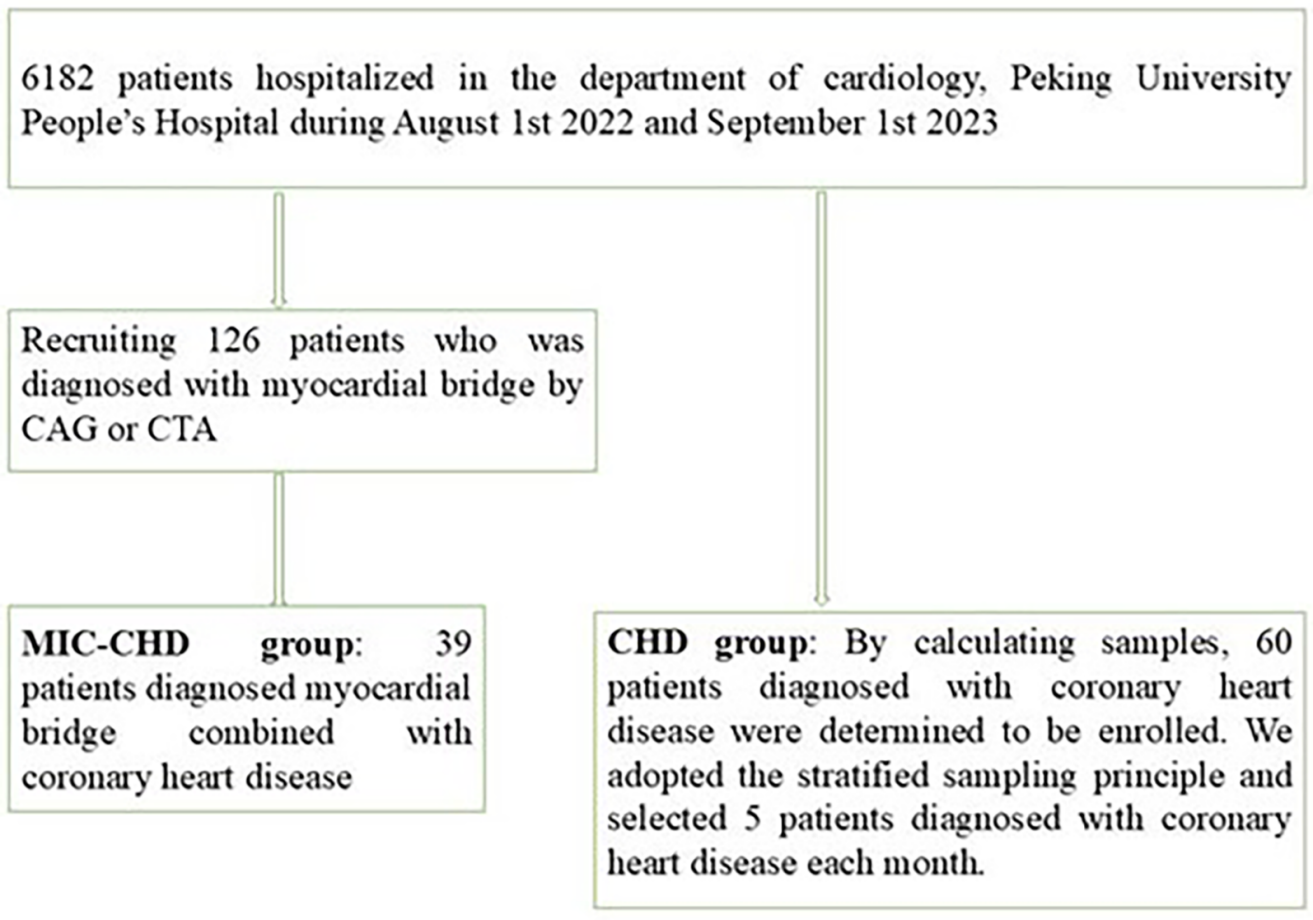
Flowchart describing study inclusion.

Study participants were recruited from the Department of Cardiology, Peking University People's Hospital from August 2022 to September 2023 (Figure 1). All patients were diagnosed utilizing CAG or CTA. The inclusion criteria were (1) patients with myocardial ischemia symptoms such as angina pectoris and ST-T segment changes and (2) those with a glomerular filtration rate above 60 ml/min. The exclusion criteria were (1) patients with other heart diseases besides CHD, (2) patients with poor physical condition and multiple organ failure, and (3) patients with hematological disease or tumor. By screening, a total of 39 patients with MIC and CHD were recruited. According to the sample size calculation formula and morbidity, 60 CHD patients were decided. We adopted the stratified sampling principle and selected five patients diagnosed with CHD each month. None of the patients had a history of positive/negative inotropic drug use. For comparison of left ventricular ejection fraction (LVEF) between the two groups, *t*-test was used, and *p* < 0.05 was considered significant. For regression analysis, we used logistic regression analysis. In the present study, 13 cases with missing data were excluded.

### Classifying MIC

According to the Schwarz classification (2009), Type A includes patients with clinical symptoms but no objective signs of ischemia and doing well with reassurance alone, Type B includes those with objective signs of ischemia by noninvasive testing, and Type C includes those who were with altered intracoronary hemodynamics and were treated with beta-blockers or calcium-channel blockers (5). MIC CAG is characterized by “stenosis due to systolic compression,” also known as “milking phenomenon” (5). According to the degree of systolic stenosis of the mural coronary artery (MCA), MICs were divided into three grades: Grade I stenosis, systolic stenosis degree <50%; Grade II stenosis, systolic stenosis degree 50%–75%; and Grade III stenosis, systolic stenosis degree >75% (19).

### Clinical data recording

The history of hypertension, diabetes, hyperlipidemia, smoking, drinking, and serological data such as brain natriuretic peptide, alanine aminotransferase (ALT), aspartate transaminase (AST), blood urea nitrogen, blood glucose, total cholesterol (TC), triglycerides, high-density lipoprotein, low-density lipoprotein, and K^+^ levels of subjects participating was recorded in the study. The diagnosis of hypertension was based on the 2017 American Hypertension Diagnostic Criteria (20): Grade 1 hypertension, systolic blood pressure 130–139 mmHg, diastolic blood pressure 80–89 mmHg, and Grade 2 hypertension, ≥140/90 mmHg. The diagnosis of diabetes was based on the 2017 American Diabetes Association diagnostic criteria (21): gasting blood glucose ≥ 7.0 mmol/L; random blood glucose ≥ 11.1 mmol/L, and patients with diabetic symptoms—2 h postprandial blood glucose ≥ 11.1 mmol/L in the glucose tolerance test. Patients with atypical symptoms needed to repeat the test to determine the presence of diabetes according to the above criteria. The diagnosis of hyperlipidemia was based on the following: serum TC >5.2 mmol/L, triglyceride (TG) >1.7 mmol/L, and serum low-density lipoprotein cholesterol >3.64 mmol/L (22, 23).

### Statistical analysis

Statistical analyses were performed using SPSS software (version 29.0). The numerical data are expressed as the mean, and the comparison between the two groups was compared using the *t*-test. The categorical variables are expressed as number and percentage, and the comparison between the two groups was performed by the chi-square test. Logistic regression was used for multivariate analysis to screen out the potential independent influencing factors of MIC-CHD. Statistical significance was defined as two-tailed. *p* < 0.05 for all tests.

## Results

### Basic clinical characteristics of the study participants

Table 1 shows the baseline clinical characteristics of the study populations. The mean age of the MIC-CHD and CHD groups was 69.57 ± 7.5 years and 64.39 ± 11.95 years, respectively (*p* = 0.016). Other clinical characteristics, such as hyperlipidemia, diabetes mellitus, cardiac troponin I (cTnI), ALT, AST, and TG show significant differences.

**Table 1:** Baseline characteristics of both groups.

Index	MB-CHD (*n* = 39)	CHD (*n* = 60)	*p*-value
Age (years)	69.57 ± 7.5	64.39 ± 11.95	0.016
Sex (*n*, %) M	11 (28.21)	32 (53.33)	0.144
Hypertension (*n*, %)	12 (30.77)	35 (58.33)	0.058
Hyperlipidemia (*n*, %)	15 (38.46)	16 (26.67)	0.038
Diabetes (*n*, %)	3 (7.70)	20 (33.33)	0.015
Smoking (*n*, %)	8 (20.51)	22 (36.67)	0.440
Drinking (*n*, %)	6 (15,38)	9 (15)	0.538
cTnI (pg/ml)	6.94 ± 7.84	146 ± 8.58	0.049
ALT (U/L)	18.55 ± 8.48	28.3 ± 28.91	0.020
AST (U/L)	20.5 ± 4.06	27.76 ± 19.64	0.010
BUN (mmol/L)	6 ± 1.4	6.11 ± 2.42	0.427
Cr (umol/L)	73.45 ± 15.39	103.31 ± 30.71	0.146
UA (umol/L)	366.82 ± 68.82	389.85 ± 126.93	0.168
GLU (mmol/L)	5.75 ± 1.17	5.79 ± 2.02	0.460
TC (mmol/L)	3.93 ± 1.01	3.86 ± 1.02	0.390
TG (mmol/L)	1.31 ± 0.47	1.62 ± 0.82	0.052
HDL (mmol/L)	1.16 ± 0.24	3.08 ± 14.01	0.262
LDL (mmol/L)	2.25 ± 0.79	2.19 ± 0.78	0.400
K^+^ (mmol/L)	4.09 ± 0.42	4.12 ± 0.44	0.398

Continuous data are expressed as mean ± SD; categorical data are expressed as frequencies. LVEF, left ventricular ejection fraction; TnI, cardiac troponin I; ALT, alanine aminotransferase; AST, aspartate transaminase; BUN, blood urea nitrogen; Cr, creatinine; UA, uric acid; GLU, glucose; TC, serum total cholesterol; TG, triglyceride; HDL, high-density lipoprotein; LDL, low-density lipoprotein cholesterol; K^+^, serum K^+^.

### Diabetes mellitus is an independent risk factor of MIC-CHD, and hyperlipidemia is an independent protective factor

Table 2 demonstrates the multi-factor regression analysis. Combined with the results of the single-factor analysis, a multifactor regression analysis was performed for the MIC-CHD and CHD groups (1, MIC-CHD group; 0, CHD group). According to the result, hyperlipidemia is an independent protective factor (*p* = 0.020, OR = 0.163, 95% CI 0.035–0754), and diabetes mellitus is an independent risk factor of MIC-CHD (*p* = 0.031, OR = 6.353, 95% CI 1.179–34.221).

**Table 2:** Univariate linear regression analysis.

Index	*p*-value	OR	95% CI	
Age (years)	0.177	1.064	0.972	1.165
Hypertension (*n*)	0.378	2.055	0.415	10.180
Hyperlipidemia (*n*)	0.020	0.163	0.035	0.754
Diabetes (*n*)	0.031	6.353	1.179	34.221
TnI (pg/ml)	0.256	0.956	0.884	1.033
ALT (U/L)	0.645	1.019	0.94	1.106
AST (U/L)	0.149	0.899	0.778	1.039
TG (mmol/L)	0.269	0.431	0.097	1.920

BNP, brain natriuretic peptide; ALT, alanine aminotransferase; AST, aspartate transaminase; BUN, blood urea nitrogen.

### MIC may reduce LVEF in patients with CHD

**Figure 2:**
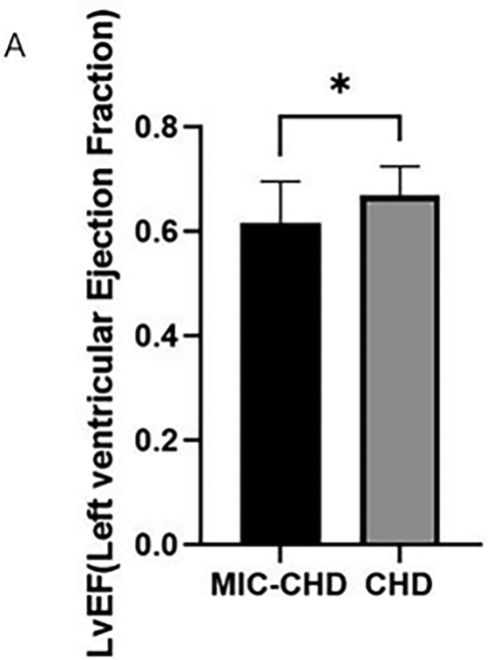
Comparison of LVEF between the MIC-CHD and CHD groups.

To observe whether MIC affected the LVEF of patients with CHD, we compared ejection fraction between the MIC-CHD and CHD groups. Figure 2 demonstrates the result. As the results show, the level of LVEF in the MIC-CHD group is lower than that of the CHD group. There is a significant difference between the two groups (*p* = 0.0153, MeanMIC-CHD = 0.62 vs. MeanCHD = 0.67), suggesting MIC may reduce ejection fraction in patients with CHD.

### In contrast to single culprit vessel, MIC has a greater effect on the cardiac function of CHD patients with more than double culprit vessels

**Figure 3:**
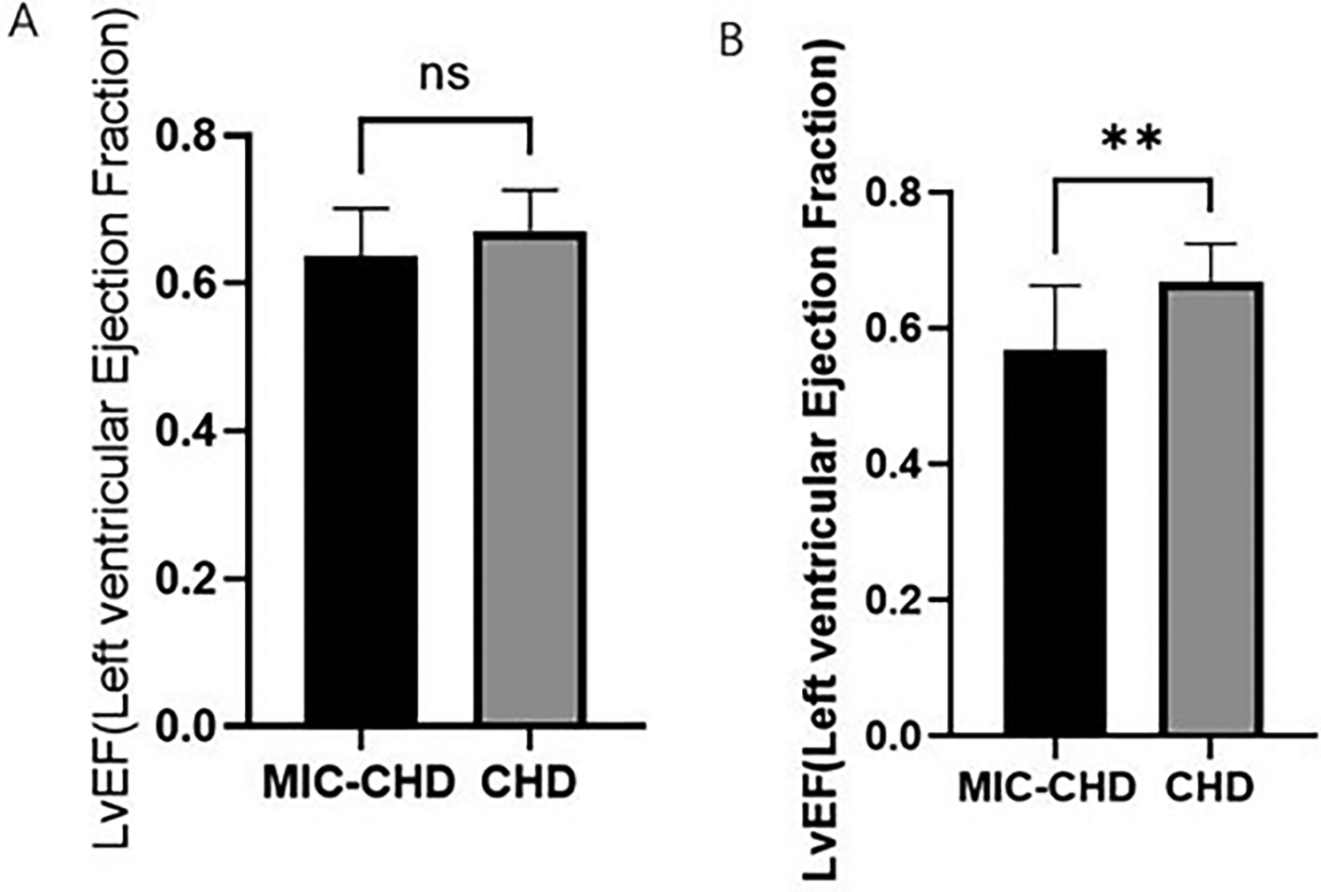
To compare the influence of myocardial intramural course on ejection fraction of different criminal vessel counts in MIC-CHD patients. **(A)** Comparison of LVEF in single branch lesions between the MIC-CHD and CHD groups (*p* = 0.3673; LVEF 0.64 vs. 0.67, respectively). **(B)** Comparison of LVEF in lesions with more than two branches between the MIC-CHD and CHD groups (*p* = 0.0019; LVEF 0.57 vs. 0.67, respectively).

By comparing LVEF between the MIC-CHD and CHD groups, MIC may reduce the cardiac function of patients with CHD. Then further observation was carried out to conclude MIC's effect on different culprit vessel numbers. Using coronary angiography, 69.6% of MIC-CHD patients had single-vessel disease, and 30.4% had more than double-vessel disease. In addition, 28.3% of CHD patients had single-vessel disease, and 71.7% had more than double-vessel disease. According to culprit vessel number, patients in both groups were divided into single-vessel disease and more than double-vessel disease separately. LVEF was compared. Figure 3A demonstrates there is no significance in single-vessel disease (*p* = 0.3673, LVEF 0.64 vs. 0.67), and Figure 3B shows a significant difference in more than double-vessel disease (*p* = 0.0019, LVEF 0.57 vs. 0.67), indicating MIC has a greater effect on the cardiac function of CHD patients with more than double culprit vessels.

### MCA length and systolic stenosis degree are inversely related to LVEF in the MIC-CHD group

**Figure 4:**
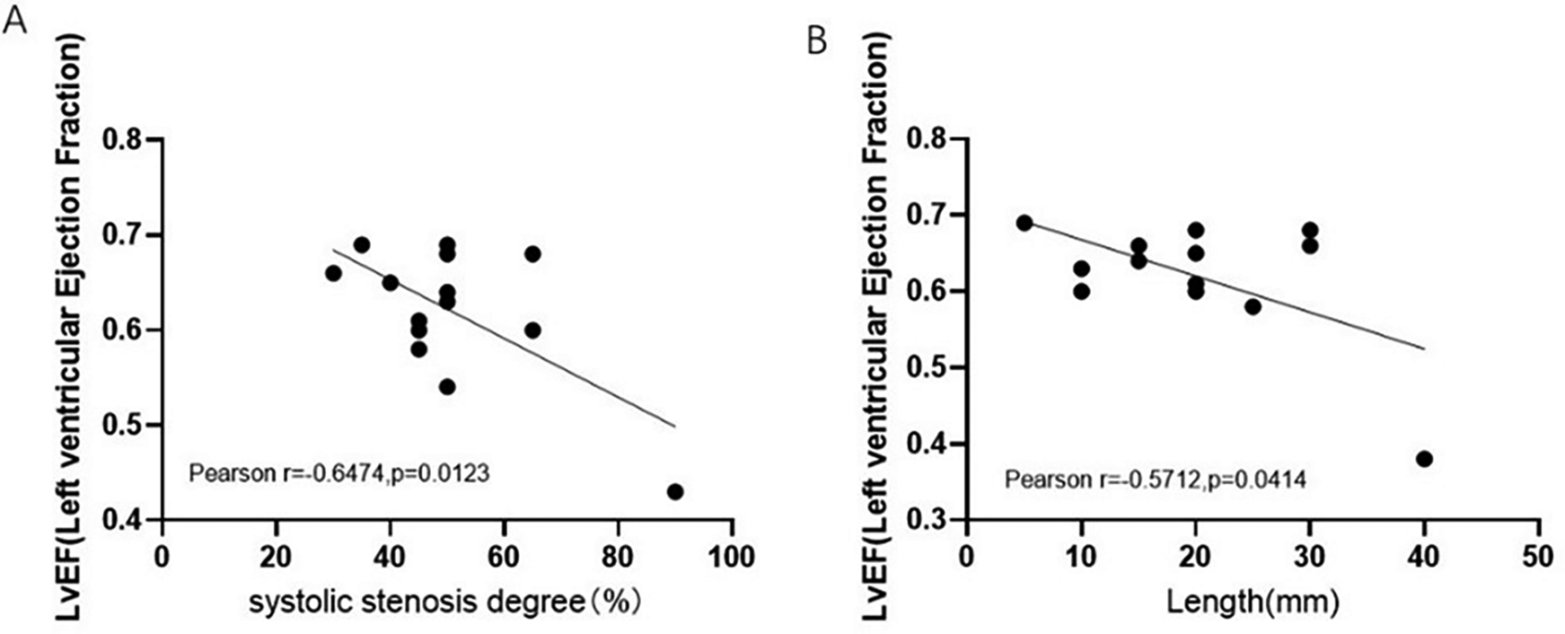
Correlation between MIC imaging index and LVEF of MIC-CHD patients. **(A)** Relation between MIC systolic stenosis degree and LVEF of patients with MIC-CHD. **(B)** The correlation between MCA length LVEF of patients with MIC-CHD. LVEF, left ventricular ejection fraction.

Owing to the milking effect, the blood supply to the heart muscle may be affected. Based on the phenomenon, imaging indexes of MIC were measured to evaluate whether MCA length and MIC systolic stenosis degree impact LVEF in the MIC-CHD group. Figure 4A presents the correlation between MIC systolic stenosis degree and LVEF of patients with MIC-CHD denoting that there is a negative correlation (*r* = −0.6474, *p* = 0.0123). The correlation between MCA length and LVEF of patients with MIC-CHD was displayed in Figure 4B, which signifies a negative relation (*r* = −0.5712, *p* = 0.0414). Our data revealed that the longer and narrower MIC a CHD patient has, the lower LVEF they may have, indicating that the longer and deeper the wall coronary artery runs in the myocardium, the more the blood supply to the heart is affected. Then patients with CHD are more likely to have symptoms of myocardial ischemia such as angina pectoris.

## Conclusion

The combination of MIC in patients with CHD may contribute to the reduction of LVEF, whereas MIC length and the systolic stenosis degree of MCA were negatively correlated with LVEF.

## Discussion

A few studies have reported that some patients with sudden cardiac death did not die from myocardial infarction caused by coronary atherosclerosis, but rather related to MIC (24–27). MICs compress coronary arteries during the systolic period of the heart cycle, causing angina pectoris, myocardial infarction, paroxysmal atrioventricular block, myocardial ischemia during exertion, and sudden cardiac death (28). With the increasing development of imaging diagnostic technology, the detection rate of myocardial ischemia caused by MIC has increased (29, 30). CTA has been widely used in the detection of MIC, and it has been proven to non-invasively measure the length and thickness of MIC, the systolic and diastolic pressure on MCA, and other MIC anatomical features (31). However, there is still a lack of research on the effect of MIC on cardiac function in patients with CHD. Therefore, we recruited patients with CHD to assess whether MIC affected cardiac function in patients with CHD. LVEF is one of the indexes used to evaluate cardiac function in the clinic. In our case–control study, we focused on the LVEF of MIC-CHD patients. We not only compared the effects of MIC cardiac function in patients with CHD between the MIC-CHD and CHD groups but also made a comparison within the MIC-CHD group to compare the effect of MIC on ejection fraction of CHD patients with different lesion counts. In previous studies, it was believed that MIC was a benign anatomical abnormality, so it did not attract enough attention. Nonetheless, we did find that MIC may reduce ejection fraction in patients with CHD, and the greater the number of vascular lesions, the greater the influence.

To make the results more accurate, we divided all participants into two subgroups: the MIC-CHD and CHD groups. To fully observe the effect of MIC on LVEF of patients with CHD, we made a comparison of LVEF between the MIC-CHD and CHD groups. The result was that the LVEF of the MIC-CHD group was lower than that of the CHD group, suggesting for patients with CHD, the presence of MIC can reduce cardiac function. Then to observe the influence of MIC on cardiac function in CHD patients with different numbers of diseased vessels, according to the number of lesions, the two groups of patients were separately divided into the single-vessel subgroup and more than double-vessel subgroup, and the ejection fraction of the two subgroups with single-vessel lesion and more than two vessel lesion was compared. Results demonstrated no significance in single-vessel disease and a significant difference in more than double-vessel disease, indicating MIC may have a greater effect on the cardiac function of CHD patients with more culprit vessels. Then we researched how the imaging index of MIC influenced on LVEF of MIC-CHD patients. We measured the MCA length and systolic stenosis degree of MCA making a comparison within the MIC-CHD group. The results showed that both the MCA length and MCA systolic stenosis degree were negatively correlated with LVEF.

The mechanism of the effect of MIC on the cardiac function of CHD patients may be as follows:

1. Limited coronary blood flow

Systolic stenosis: When the heart contracts, the heart muscle covering the coronary arteries compresses the arteries as the heart contracts, causing the coronary lumen to narrow, forming a systolic stenosis. This narrowing restricts blood flow to the coronary arteries, reducing the supply of blood to the heart during contraction.

Diastolic relaxation competes with blood flow: When the heart diastoles, the cardiac bridge relaxes and the coronary lumen returns to normal, forming diastolic relaxation. However, at this time, the blood flow of the wall coronary artery is subject to competition from both ends of the MIC, because the coronary artery at both ends of the MIC has a larger diameter and more blood flow, while the wall coronary artery has relatively less blood flow, resulting in insufficient diastolic blood flow.

2. Myocardial ischemia and functional impairment

Myocardial ischemia: Because coronary blood flow is restricted during both systolic and diastolic periods, the heart muscle does not get enough oxygen and nutrients, resulting in myocardial ischemia. The severity of myocardial ischemia depends on the length, depth, location, thickness, elasticity, and other factors of the MIC, as well as the coronary blood flow reserve, vasospasm, atherosclerosis, and other factors.

Impaired function: Myocardial ischemia can lead to impaired myocardial function, manifested as reduced left ventricular diastolic function, left ventricular systolic function, and local contraction deformation. In addition, myocardial ischemia may also cause serious complications such as arrhythmia and heart failure.

3. Pathophysiological mechanism

Decreased wall coronary artery flow reserve: MIC compression of wall coronary artery continued to the early and middle diastolic period, resulting in a temporary decrease in wall coronary artery flow reserve, which in turn affected ventricular diastolic function.

Atherosclerosis and vascular endothelial damage: Areas of vascular shear and blood flow deviation from the laminar unidirectional pattern near the MIC (including areas of oscillating blood flow and blood flow reversal) can promote atherosclerotic plaque formation, leading to endothelial dysfunction. In addition, the MIC repeatedly compresses and distorts the wall of coronary arteries, which may also lead to coronary spasms and vascular endothelial damage.

Myocardial fibrosis: Chronic myocardial ischemia caused by MIC contraction may aggravate myocardial fibrosis and further aggravate ventricular mechanical and electrical remodeling, inducing adverse cardiac events. In addition to reducing myocardial oxygen consumption by taking drugs such as beta-blockers, it may be possible to increase the utilization of oxygen by the heart muscle through drugs.

The formation of MIC is considered a congenital abnormality (32). Then further research may focus on whether such patients have genetic abnormalities or abnormal gene expression. Moreover, serological markers should be found to make a timely diagnosis. We believe that in the future, it may be possible to find the changed genes and proteins in patients with MIC through second-generation sequencing and proteomics so that they can be used as markers of MIC. Because MCA is nobbed by myocardial fibers, it is bound to cause changes in MCA hemodynamics, and in this case, cells or tissues may produce some substances secreted into the blood. If these substances can be found, then MIC can be diagnosed by a simpler method, and consequently, the occurrence of CHD caused by MIC may be lessened.

Due to its limited sample size and single-center research, the results of this study are unable to be used as clinical criteria currently. In addition, the limitation of this study is that no other anatomical features of MIC, such as MIC thickness or MIC location, were observed. The thickness of the MIC is a key factor in determining the degree of compression of the coronary artery. In general, the thicker the myocardium bridge, the heavier the pressure on the coronary arteries, potentially leading to more serious heart problems. Light myocardium bridge: If the thickness of the myocardium bridge is light, such as the superficial myocardium bridge (the myocardium thickness is generally not >2 mm), its compression of the coronary artery is relatively light. Patients with this type of cardiac bridge may have no obvious symptoms, or occasionally experience chest tightness, but usually resolve on their own. Heavy MIC: When the thickness of the MIC exceeds a certain limit (such as >2.5 mm), its compression of the coronary artery will become more obvious. This may lead to chest tightness, chest pain, palpitations, shortness of breath, and other symptoms and in severe cases may also cause myocardial infarction, heart failure, and other diseases. Deep myocardial intramural courses (myocardium thickness >2 mm) compress the coronary artery more heavily, and patients are more prone to clinical symptoms. The location of the MIC is also an important factor affecting cardiac function. Myocardial intramural courses are mainly located in the left anterior descending branch, most commonly in the near and middle of the left anterior descending branch (accounting for 67%–98% of the number of myocardial intramural courses), while the right coronary artery and left circumflex branch are less common. Anterior descending branch MIC: Since the anterior descending branch is one of the main branches of the coronary artery, it is responsible for providing blood supply to most of the heart muscle in the left ventricle. Therefore, the MIC located in the anterior descending branch has a more significant effect on cardiac function. We suggest that a multicenter and large-scale study should be performed to obtain more effective indicators to improve patients' life quality and better prevent CHD. In conclusion, our study found that MIC may reduce LVEF in patients with MIC-CHD, especially for CHD patients with multivessel disease, to whom it has a greater impact. The MCA length and systolic stenosis degree are negatively correlated with LVEF.

## Data Availability

The original contributions presented in the study are included in the article/Supplementary Material, further inquiries can be directed to the corresponding authors.
